# From Juvenile Idiopathic Arthritis to Pachydermoperiostosis: A Journey to an Unexpected Rare Diagnosis

**DOI:** 10.3390/jcm15030956

**Published:** 2026-01-24

**Authors:** Marilena Stoian, Ana Maria Dumitrescu, Claudia Ciofu, Bogdan Gavrila

**Affiliations:** 1 Internal Medicine and Rheumatology Department, Dr I. Cantacuzino Hospital, Carol Davila University of Medicine and Pharmacy, 020475 Bucharest, Romania; marinela.stoian@umfcd.ro (M.S.); claudia.ciofu@umfcd.ro (C.C.); bogdan.gavrila@umfcd.ro (B.G.); 2 Dclinic, 013738 Bucharest, Romania

**Keywords:** pachydermoperiostosis, primary hypertrophic osteoarthropathy, juvenile idiopathic arthritis

## Abstract

Pachydermoperiostosis, also known as primary hypertrophic osteoarthropathy or Touraine–Solente–Golé syndrome, is a rare genetic disorder that represents a small fraction of hypertrophic osteoarthropathy cases. It typically begins during adolescence, affects males more frequently and follows either an autosomal dominant or recessive inheritance pattern. The disease is characterized by the triad of pachydermia, periostosis and digital clubbing, often accompanied by hyperhidrosis, seborrhea, cutis verticis gyrata and joint effusions. Although articular involvement is usually non-erosive, the disorder may mimic inflammatory arthritis and lead to diagnostic delays. Recognition of the major and minor diagnostic criteria is crucial to distinguish PDP from secondary forms related to pulmonary, cardiac or neoplastic disease.

## 1. Introduction

Pachydermoperiostosis (PDP), also known as Primary Hypertrophic Osteoarthropathy (PHO) or Touraine–Solente–Golé syndrome, is a rare hereditary disorder. It represents the idiopathic form of hypertrophic osteoarthropathy and may follow either an autosomal dominant or recessive inheritance pattern. Expression is highly variable, even within the same family, which often makes diagnosis more challenging [1,2].

Hypertrophic osteoarthropathy is usually divided into two groups. The primary form, which corresponds to PDP, is relatively uncommon. By contrast, the secondary form occurs much more frequently and usually develops in association with chronic pulmonary or cardiac conditions, as well as certain gastrointestinal diseases and malignancies [1,3].

From an epidemiological perspective, PDP is extremely uncommon, with prevalence estimated around 0.16% in some studied populations. The disorder typically begins during adolescence and shows a clear male predominance, with men affected more often and more severely than women; the reported sex ratios are close to 7:1. Despite its rarity, more than 200 cases have been documented in the literature, many occurring within familial clusters, supporting its strong genetic background [1,4,5].

Clinically, PDP is distinguished by a triad of findings: thickened skin of the face and scalp (pachydermia), enlargement of the distal digits (clubbing) and excessive sweating (hyperhidrosis). Other cutaneous features such as seborrhea or acne may also be observed, while skeletal involvement contributes to enlargement of the extremities. Depending on how these features are combined, three clinical variants have been recognized:

- a complete form, where both skin and bone changes are present;
- an incomplete form, in which periostosis and clubbing occur without pachydermia;
- a forme fruste, where pachydermia predominates and skeletal modifications are minimal or absent [1,6,7].

At the molecular level, the condition is linked to disturbances in prostaglandin E_2_ (PGE_2_) metabolism. Mutations in the 15-hydroxyprostaglandin dehydrogenase gene (HPGD) or solute carrier organic anion transporter family member 2A1 (SLCO2A1) genes impair prostaglandin degradation and transport, resulting in persistently elevated PGE_2_ levels. This biochemical imbalance drives periosteal bone formation, vascular proliferation and skin thickening with hyperhidrosis [1,2,6].

In contrast with secondary hypertrophic osteoarthropathy, PDP usually starts earlier in life, most often during puberty, progresses gradually and demonstrates a clear hereditary pattern [1,3].

## 2. Case Presentation

We present the case of a 19-year-old male patient admitted to our clinic in April 2024, referred by his family physician, for polyarticular swelling (predominantly knees), without pain, associated with ankle hyperhidrosis and marked skin folds on the forehead, together with facial seborrhea.

His family history was not notable, with no major diseases and no similar illness among relatives. The patient is a non-smoker and does not consume alcohol.

Regarding his past medical history, we note a diagnosis of polyarticular juvenile idiopathic arthritis–rheumatoid factor (RF) negative (polyarticular JIA RF negative-2021) according to the ILAR (International League of Associations for Rheumatology), initially treated with methotrexate (MTX-15 mg/week) for approximately 6 months, and discontinued due to gastrointestinal intolerance. It should also be noted that anti-citrullinated protein antibodies (anti-CCP) were negative and also the HLA B27 antigen. Unfortunately, for various reasons, the patient says that he did not present for further rheumatology evaluations.

In 2024, the patient reported, in addition to articular swellings, the new onset of cutaneous manifestations including furrowing of the forehead and cheeks, seborrhea, eyelid edema, clubbing and distal skin thickening.

**Figure 1:**
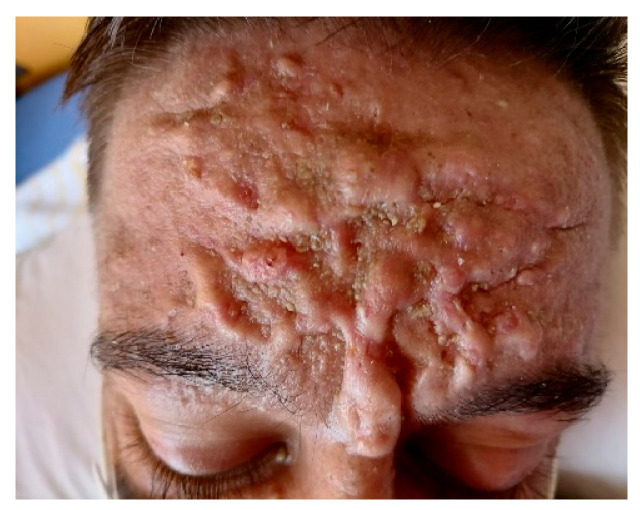
Marked skin folds, furrowing of the skin and keloid skin.

Clinical examination revealed marked forehead folds, furrowing of the forehead and cheeks, keloid skin, facial seborrhea and eyelid edema (Figure 1).

**Figure 2:**
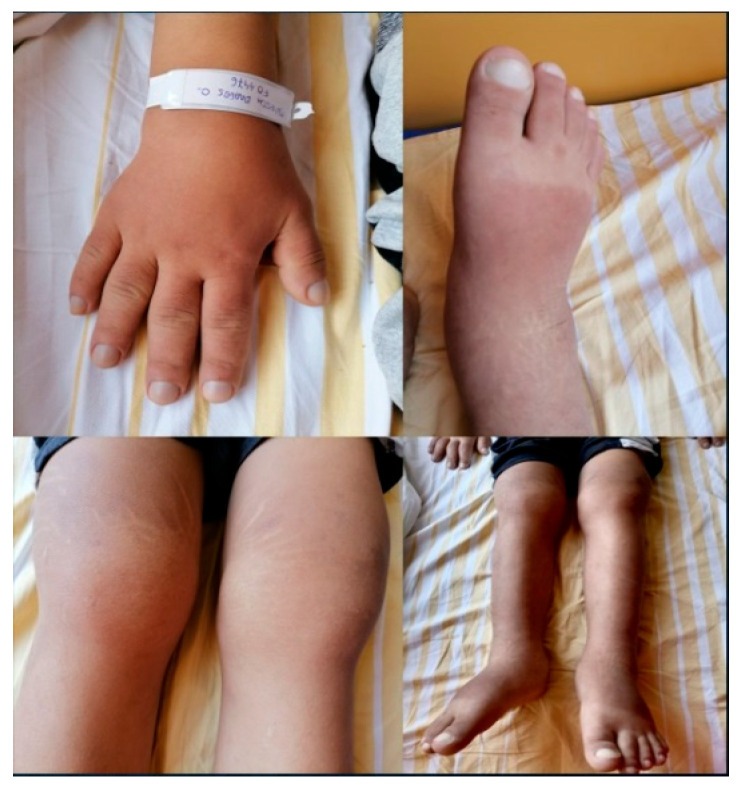
Enlargement of distal extremities and digital clubbing.

Additional findings included distal skin thickening of the forearms, arms, legs and feet; enlargement of distal extremities; digital clubbing of both fingers and toes; bilateral knee effusions without pain (Figure 2) and ankle hyperhidrosis. The rest of the general clinical examination was without particularities.

Laboratory findings: mild normocytic normochromic anemia (Hb 10.4 g/dL), elevated erythrocyte sedimentation rate (ESR 61 mm/h, N < 30) and C-reactive protein (CRP 62 mg/L, N < 5). Iron deficiency was noted (serum iron 35 µg/dL, N 59–158) with increased ferritin (223 ng/mL, N 15–150) and hypergammaglobulinemia (IgG 2910 mg/dL), with no paraprotein founded after performing serum protein electrophoresis with immunofixation.

The autoimmune profile was negative (antinuclear antibodies—ANA, RF and anti-CCP). Liver, kidney and thyroid function tests were normal. Tumor markers were negative. Insulin-like growth factor 1 (IGF-1) and the oral glucose tolerance test were within normal limits, excluding acromegaly.

**Figure 3:**
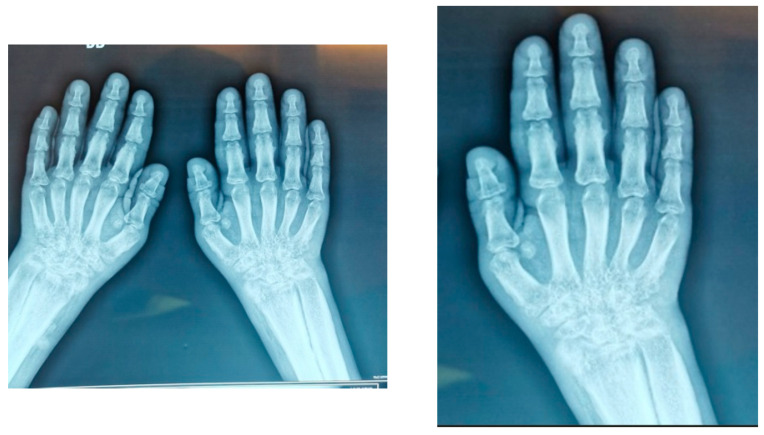
Hand X-rays.

**Figure 4:**
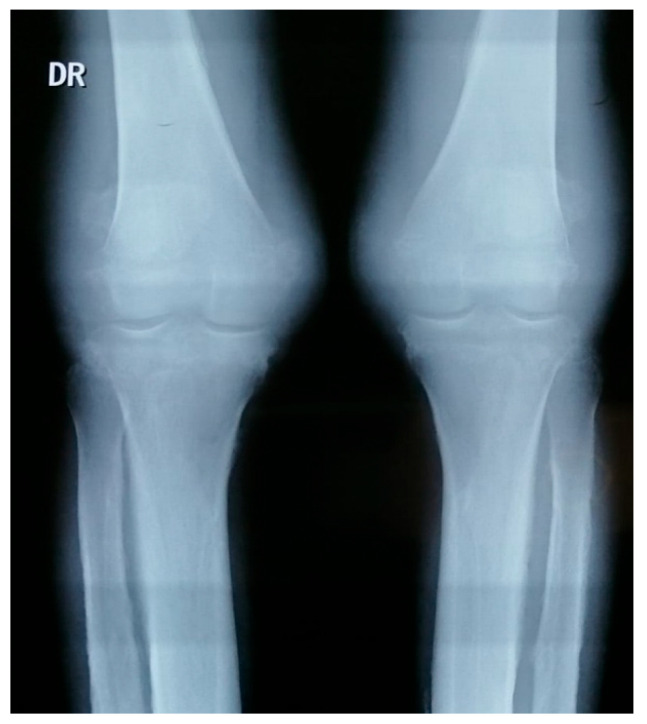
Knee X-rays.

**Figure 5:**
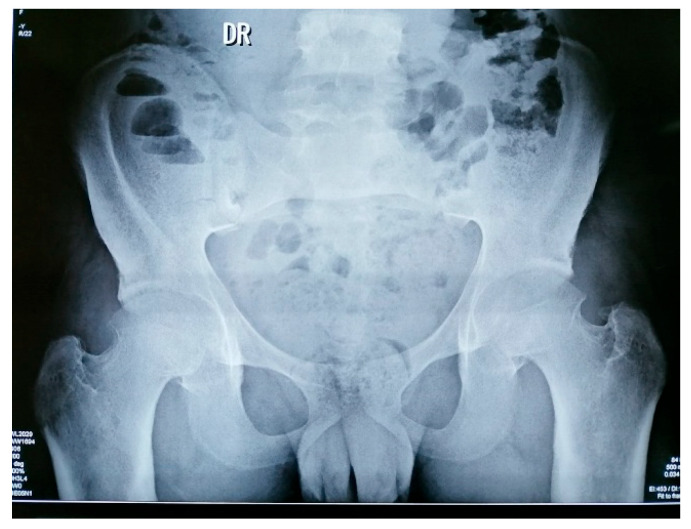
Pelvis X-rays.

Imaging:

- Hand X-rays: bilateral carpitis with cortical thickening (Figure 3)
- Knee X-rays: periosteal reaction involving tibia, fibula and femur (Figure 4).
- Pelvis X-rays: acetabular osteosclerosis, bilateral reduction in coxofemoral joint space and thickened proximal femurs (Figure 5)
- CT thorax/abdomen: pericardial effusion (6.2 mm), bilateral gynecomastia, hepatomegaly with microbiliary cyst (segment VIII) and renal microlithiasis.

**Figure 6:**
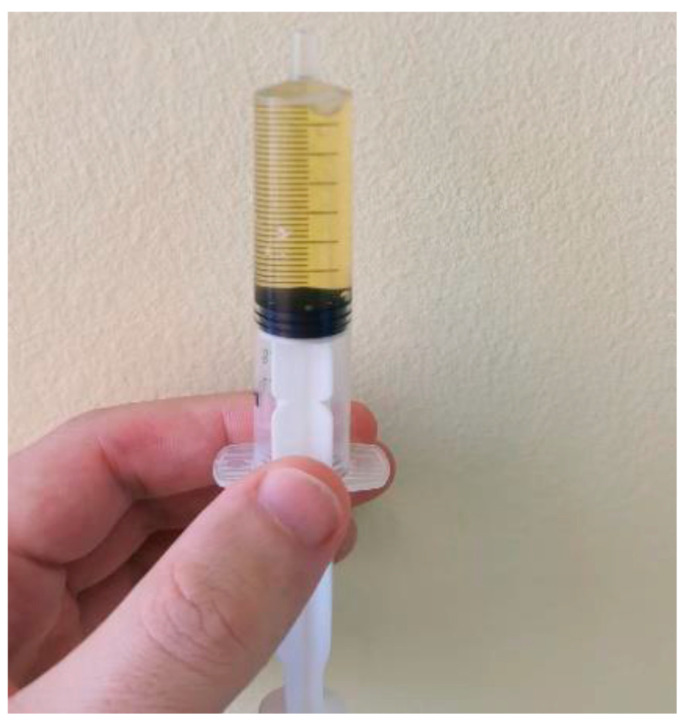
Joint fluid aspiration.

Arthrocentesis: diagnostic and therapeutic joint aspiration was performed, with extraction of 260 mL serocitrinous fluid from each knee (total 520 mL), followed by intra-articular administration of betamethasone. Bacteriological and biochemical analysis: sterile fluid, glucose within normal range and RF absent. Cytology: poor smear (<200 cells/mm^3^), PMN < 25%, rare red blood cells; no neutrophils, granulocytes, synovial cells or ragocytes (Figure 6).

Endocrinological evaluation: hormone panel included estradiol, FSH, LH, prolactin, testosterone, hGH and IGF-1 (somatomedin C) with normal values.

**Figure 7:**
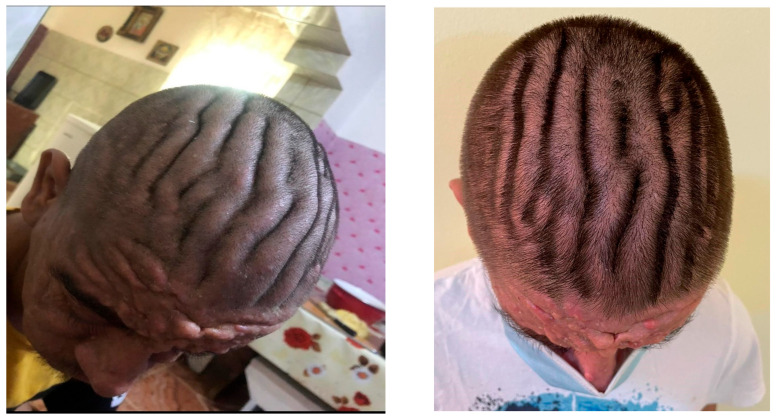
Scalp cutis verticis gyrata.

November 2025 follow-up: new findings included thick transverse skin folds of the scalp consistent with cutis verticis gyrata, blepharoptosis (Figure 7) and worsening ankle swelling.

Final diagnosis was made complete form of pachydermoperiostosis (Touraine–Solente–Golé syndrome), bilateral knee arthritis, idiopathic pericarditis, gynecomastia and mild anemia in an inflammatory context.

Treatment: methylprednisolone 16 mg/day, colchicine 1 mg/day, sulfasalazine 3 g/day, vitamin D supplementation, potassium supplements and omeprazole. Orthopedic evaluation considered synovectomy for persistent effusions.

Unfortunately, the patient missed his physical appointments to assess the clinical and biological evolution under the prescribed treatment.

## 3. Discussion

Our patient exhibited the complete form of pachydermoperiostosis, characterized by pachydermia, periostosis and digital clubbing, together with several minor features such as hyperhidrosis, blepharoptosis, seborrhea, joint effusions and cutis verticis gyrata. Systemic involvement, including pericardial effusion and gynecomastia, further underlined the multisystemic nature of the disease [8,9].

One notable finding in this case was the presence of carpal joint space narrowing with destructive changes, features that are not typically described in primary hypertrophic osteoarthropathy. The articular manifestations of pachydermoperiostosis usually consist of arthralgia, non-erosive joint effusions and periosteal bone formation, particularly at the knees and ankles [8,10]. Erosive arthritis and severe joint space loss are considered exceptional, with only rare reports involving large joints such as the hips [11,12]. Therefore, the destructive carpitis observed in our patient likely reflects an overlapping inflammatory process, possibly related to his prior diagnosis of juvenile idiopathic arthritis, rather than a direct manifestation of pachydermoperiostosis itself.

The diagnostic framework was guided by the recognition of the major criteria (pachydermia, periostosis and digital clubbing) and multiple minor criteria, which clearly supported the diagnosis of the complete form of pachydermoperiostosis [9,10]. The coexistence of atypical carpal involvement emphasizes the need for careful differential diagnosis, including juvenile idiopathic arthritis, rheumatoid arthritis, psoriatic arthritis and SAPHO syndrome [8,9].

Therapeutic options remain symptomatic, focusing on anti-inflammatory drugs, corticosteroids, colchicine and in selected cases, bisphosphonates or COX-2 inhibitors [10,13]. Retinoids may help with seborrhea, while surgical procedures such as synovectomy or plastic surgery are considered in refractory cases. In our patient, we considered also giving colchicine due to pericarditis, since some clinical cases in the literature showed improvement of joint and bone inflammation after treatment with colchicine [14].

This case underlines the importance of applying clinical diagnostic criteria and maintaining a broad differential diagnosis when evaluating young male patients with atypical articular swelling, especially when cutaneous changes and systemic manifestations are present. A multidisciplinary approach is essential for optimal management [8,14].

## 4. Conclusions

This case highlights the complexity of diagnosing pachydermoperiostosis, particularly in the setting of a previous diagnosis of juvenile idiopathic arthritis. The patient fulfilled all three major criteria and several minor features, supporting the diagnosis of the complete form of the disease [9,10].

The observation of destructive carpal involvement is atypical for pachydermoperiostosis and may indicate coexisting inflammatory arthritis rather than a direct expression of the disorder [11,12,14]. This underscores the importance of considering overlapping conditions when evaluating patients with unusual articular patterns.

Accurate recognition of pachydermoperiostosis based on its defining clinical triad prevented unnecessary prolonged immunosuppression and allowed for targeted symptomatic management. Early diagnosis and a multidisciplinary approach are essential, while further research is needed to clarify the mechanisms underlying possible overlap between pachydermoperiostosis and inflammatory arthritis [9,14].

## Data Availability

The original contributions presented in this study are included in the article. Further inquiries can be directed to the corresponding author(s).

## Abbreviations

The following abbreviations are used in this manuscript:

PDP	Pachydermoperiostosis
PHO	Primary hypertrophic osteoarthropathy
